# Therapist-Assisted Progressive Resistance Training, Protein Supplements, and Testosterone Injections in Frail Older Men with Testosterone Deficiency: Protocol for a Randomized Placebo-Controlled Trial

**DOI:** 10.2196/resprot.8854

**Published:** 2018-03-02

**Authors:** Rune Rasmussen, Mette Midttun, Tine Kolenda, Anne-Mette Ragle, Thea Winther Sørensen, Anders Vinther, Bo Zerahn, Maria Pedersen, Karsten Overgaard

**Affiliations:** ^1^ Department of Neurology University Hospital of Copenhagen Herlev Hospital Herlev Denmark; ^2^ Medical Department O University Hospital of Copenhagen Herlev Hospital Herlev Denmark; ^3^ Department of Rehabilitation University Hospital of Copenhagen Herlev Hospital Herlev Denmark; ^4^ Department of Clinical Physiology and Nuclear Medicine University Hospital of Copenhagen Herlev Hospital Herlev Denmark

**Keywords:** accidental falls, aged, exercise, testosterone, therapeutics, men

## Abstract

**Background:**

Fall accidents are a major cause of mortality among the elderly and the leading cause of traumatic brain injury. After a fall, many elderly people never completely recover and need help in coping with everyday life. Due to the increasing older population in the world, injuries, disabilities, and deaths caused by falls are a growing worldwide problem.

Muscle weakness leads to greatly increased risk of falling, decreased quality of life, and decline in functional capacity. Muscle mass and muscle power decrease about 40% from age 20 to 80 years, and the level of testosterone decreases with age and leads to impaired muscle mass. In addition, 20% of men older than 60 years—and 50% older than 80 years—have low levels of testosterone. Treatments after a fall are significant financial burdens on health and social care, and it is important to find treatments that can enhance function in the elderly people.

**Objective:**

The purpose of this study is to investigate whether testosterone and progressive resistance training alone or combined can improve muscle strength and reduce the risk of falls in older men. Additionally, we will examine whether such treatments can improve quality of life, functional capacity, including sexual function, and counteract depression.

**Methods:**

This is a randomized placebo-controlled, double-blind trial in which frail older men with testosterone deficiency are treated with testosterone supplemental therapy and therapist-assisted progressive resistance training for 20 weeks, with the possibility to continue treatment for 1 year. Four study arms of 48 participants each are provided based on factorial assignment to testosterone supplemental therapy and progressive resistance training. The 4 groups are as follows: controls given placebo injections without physical exercise for 20 weeks, testosterone-alone group given testosterone injections without physical exercise for 20 weeks, training-alone group given placebo injections for 20 weeks combined with 16 weeks of progressive strength training, and combination group given testosterone injections for 20 weeks combined with 16 weeks of progressive strength training.

Performance in the 30-second chair stand test to measure improvement of general strength, balance, and power in lower extremities is the primary endpoint. Secondary endpoints comprising tests of cognition, muscle strength, and quality of life are applied before and after the training.

**Results:**

Funding was provided in October 2016. Results are expected to be available in 2020. Sample size was calculated to 152 participants divided into 4 equal-sized groups. Due to age, difficulty in transport, and the time-consuming intervention, up to 25% dropouts are expected; thus, we aim to include at least 192 participants.

**Conclusions:**

This investigation will evaluate the efficacy of testosterone supplemental therapy alone or combined with progressive resistance training. Additionally, improvements in quality of life and cognition are explored.

**Trial Registration:**

Clinicaltrials.gov NCT02873559; https://clinicaltrials.gov/ct2/show/NCT02873559 (Archived by WebCite at http://www.webcitation.org/6x0BhU2p3)

## Introduction

Among the elderly, invalidity and mortality after falling constitute a major health problem. Every year, about 40% of all people aged 65 years or older experience a fall, and approximately 10% of these falls lead to serious injury and increased mortality and invalidity [1-3]. Invalidity after fall accidents increases the need for support and is associated with extensive social costs expected to rise significantly in the future [4-6].

### Accidental Falls in the Elderly: Causes and Injuries

The cause of serious falls is because of several factors: primarily reduced muscle strength in the elderly, especially in the thigh muscles [7]. Muscle mass decreases by about 40% from age 20 to 80 years [7].

Specifically, the loss of muscle power (muscle strength × contraction rate) associated with sarcopenia is related to increased risk of falling and decreases by approximately 3.5% annually from the age of 65 years [8]. In addition, 1 week of immobilization may result in a reduction of muscle strength of up to 20%, and a loss of bone of up to 1% of the maximum bone mass corresponding to the normal annual reduction [9]. Especially among weak elderly persons, extended bed rest can cause muscle strength to decline below a critical threshold because of which basic daily activities can no longer be performed. However, significant gains in both muscle strength and muscle power can be achieved through strength training in the elderly. In a 2009 Cochrane review of 121 studies, investigators found that 8 to 12 weeks of progressive strength training significantly increased muscle strength by 10% to 45% in persons aged 60 years and older [7].

Male testosterone levels decrease with age. Furthermore, 20% of men older than 60 years and 50% older than 80 years are hypogonadal with serum testosterone below 10 nmol/L or 300 ng/dL [10,11]. The normal average is approximately 22 nmol/L (650 ng/dL) with an upper limit of 35 nmol/L (1000 ng/dL) [11].

### Hypogonadism

Hypogonadism is associated with impaired muscle mass, muscle strength, and bone mass [10,12]. In a meta-analysis of 17 studies, 3 to 36 months of testosterone supplement induced a significant increase of 2.7% in lean body mass (corresponding to increased muscle mass) [12]. Despite significant increases in muscle mass, only tendencies toward increases in muscle strength were observed in two meta-analyses of, respectively, 10 and 11 studies investigating 1 to 39 months of testosterone supplementation [12,13]. Thus, it could be speculated that a training stimulus may be needed to translate the increased muscle mass into a measurable increase in muscle strength. In older men with verified hypogonadism, positive effects on bone mass have been found after 24 to 36 months of testosterone supplementation [14,15]. In a randomized controlled trial, it was shown that the growth in muscle mass, strength, and power induced by testosterone supplementation was dose dependent [16]. Testosterone supplemental therapy for hypogonadal men has resulted in a significant improvement in balance [17], and in a recent study, investigators found a direct correlation between testosterone deficiency and increased risk of fall [18].

The effect of strength training is increased when supplemented with protein intake immediately after exercise [19-22]. For many elderly persons, malnutrition is a barrier to improvements achieved by strength training [10]. Vitamin D deficiency can lead to loss of bone mass and adversely affect the neuromuscular function [23], and vitamin D supplements may reduce the risk of fall in the elderly [24]. A study of 100 resident elderly showed that 19% had moderate (12-25 nmol/L) and 12% had severe (<12 nmol/L) vitamin D deficiency [23].

Unlike previous studies, participants in this study will be older and have verified hypogonadism. By continuous regulation of protein and vitamin D supplementation, participants will be ensured optimal conditions for strength training. Testosterone supplementation has been shown to prevent impaired bone and muscle mass, and improved body composition, quality of life, and physical ability in a controlled study of the effect of testosterone without concurrent training for elderly men with low and slightly reduced testosterone levels [25]. Additional studies are needed to verify the above-mentioned results.

Hypogonadism is a risk factor for obesity, type 2 diabetes, atherosclerosis, myocardial infarction, chronic heart failure, and erectile dysfunction [26-28]. Furthermore, it has been found that testosterone supplements for hypogonadal men may reduce depression and improve cognition [29-31]. Testosterone supplements for men with testosterone deficiency significantly counteract erectile dysfunction, that is, impotence [32].

Dosage of approximately 100 mg testosterone weekly was associated with the best cognitive results, compared with significantly higher or lower doses [33]. Experiments with particularly positive effects of testosterone supplements used weekly intramuscular injections with approximately 100 mg slow-acting testosterone esters, although the effect of transdermal applications was not equivalently positive [34]. Intramuscular injections have been associated with improved bone mass, although transdermal testosterone did not induce a similar effect [35].

### Study Aim and Hypotheses

The aim of this study was to investigate the effect of intramuscular injections of testosterone and progressive resistance training either alone or in combination in older men. Our hypotheses are that both interventions can improve muscle strength and potentially reduce the risk of falls, and that an additive effect of a combined intervention will be present. Additionally, we will examine the effect on quality of life, functional capacity, sexual function, and depression. The findings of this trial may have fundamental importance for future recommendations for the elderly male population and for efforts to improve quality of life by reducing muscle weakness, loss of function, bone loss, falls, and fractures.

## Methods

### Study Design

This trial is designed as a double-blind, randomized, placebo-controlled intervention trial using a 2×2 factorial design.

### Recruitment of Study Participants

Eligible patients are consecutively recruited primarily by newspaper advertising and then included from several departments at Herlev University Hospital, including the Medical and Geriatric Departments, the Injury Center, and the Emergency Medical Reception Section. Thus, this is a single-center trial. Permission to use newspaper advertising was granted by the ethical committee. A screening log is kept. In addition, medical records from the outpatient fall clinic and the geriatric department are retrospectively screened up to 2 years before the start of the project, and potentially eligible patients are invited for eligibility screening. Anonymous information from medical records may be disclosed. Participation is optional, and participants must provide a written consent.

The first contact to an eligible trial participant is made when a participant contacts an investigator according to the instruction provided through newspaper advertising or in connection with a hospitalization at Herlev University Hospital. The contact will be made to the primary investigator, who at the hospital provides eligible participants with written trial information and oral information about the trial. The conversation takes place undisturbed, either in the single room or an office. Eligible patients will be informed of their rights, and if necessary, relatives will also be informed. Persons with pronounced dementia or severe cognitive impairment will not be included, in accordance to the exclusion criteria mentioned below. Each participant is allowed up to 1 week to provide written consent.

### Participants

The trial is intended to enroll 192 hypogonadal older men with physical impairment causing reduced walking ability and increased risk of falls. Criteria for inclusion and exclusion of participants follow the national treatment guide for male testosterone deficiency prepared by the Danish Endocrinological Society.

### Randomization and Blinding

Participants are randomized to 4 groups, each with 48 participants by opening sealed consecutive numbered envelopes, each containing a computer-generated treatment group assignment of the patient. To ensure evenly age distribution, 50% of the envelopes are for participants aged 70 to 84 years and 50% are for participants aged 85 years or older. Thus, the following 4 groups are established:

- A control group is given placebo injections without physical exercise for 20 weeks.
- A testosterone group is given testosterone injections without physical exercise for 20 weeks.
- A training group is given placebo injections for 20 weeks combined with 16 weeks of progressive strength training and supplementation of vitamin D, calcium, and protein commencing 4 weeks after the first injection.
- A combination group is given testosterone injections for 20 weeks combined with 16 weeks of progressive strength training and supplementation of vitamin D, calcium, and protein commencing 4 weeks after the first injection.

Participants are randomized into 4 arms so that the effects of training supplemented with vitamin D and protein, testosterone supplementation, or the combination of exercise and testosterone supplementation can be evaluated against placebo. Furthermore, the effect of combination treatment can be evaluated against the two individual treatments. Note that only participants in the 2 groups receiving progressive strength training are receiving supplements of vitamin D, calcium, and protein. Vitamin D, calcium, and protein are only given on days where participants receive progressive strength training corresponding to 3 times weekly.

**Figure 1:**
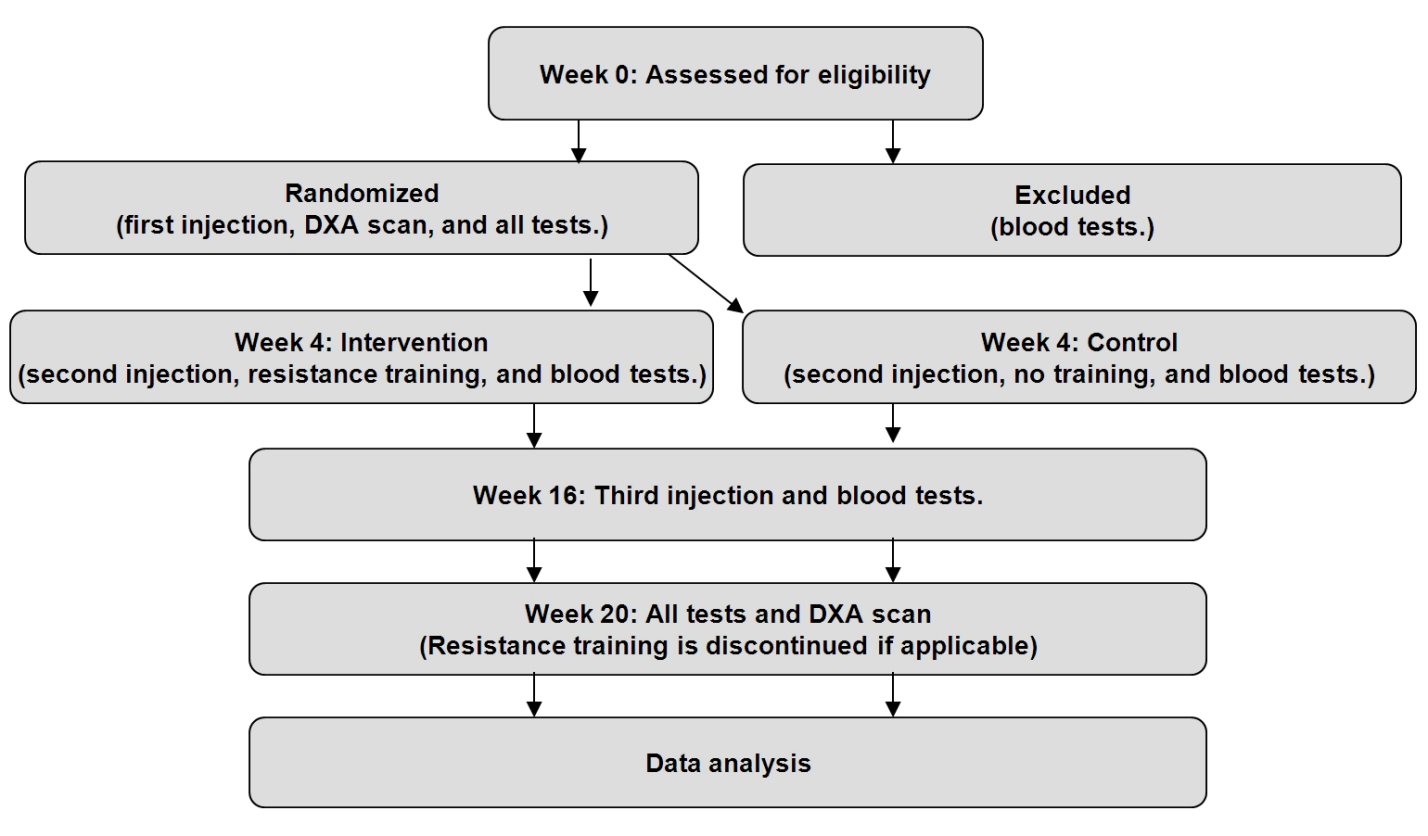
Flowchart.

The treatment with testosterone is made double blind, whereas exercise with training must be single blind, because it is not possible to blind the patients for exercise. We will ensure that investigators testing participants or performing statistical analyses are blind to treatment arms. A flowchart is given in Figure 1, showing the basic trial setup where treatments are discontinued in week 20.

During week 12, participants are asked if they are willing to continue the trial for a total of 52 weeks including injections in weeks 28 and 40 to investigate longitudinal treatment results. After week 20, such participants are asked to continue treatments according to their initial treatment arm, and participants receiving progressive resistance training are asked to continue similar training on their own. In week 52, all participants receiving extended treatment are retested using the same test battery as they completed at baseline and in week 20.

### Eligibility

Inclusion and exclusion criteria are given in Textbox 1. Note that serum testosterone level was based on a previous study [16].

### Supplements

Testosterone supplementation is given intramuscularly using 1000 mg testosterone undecanoate, which has a lasting effect of about 12 weeks [36-39], but which can be repeated more frequently between first and second administration. Therefore, the injection is repeated in week 4. A total of 3 injections are expected per subject in weeks 0, 4, and 16. Experiments have shown that injections of 1000 mg testosterone undecanoate resulted in normalization of testosterone levels in hypogonadal men without significant fluctuations. The placebo group is treated with a similar solution just without testosterone undecanoate.

**Inclusion and exclusion criteria for this study.**

Inclusion criteria

- Men aged 70 years and older experiencing loss of physical function, but who are able to walk independently with or without the use of assistive devices
- A 30-second chair stand test performance ≤12, a timed up and go performance ≥14 seconds, or walking difficulties must be present with significantly reduced walking ability and balance problems at least during the last month
- Serum testosterone level ≤13 nmol/L

Exclusion criteria

- Patients in active medical treatment for prostate cancer, or prostatic specific antigen>5 ng/mL if a urologist subsequently diagnoses a treatment-requiring prostate cancer
- Severe cardiovascular disease
- Liver aspartate transaminase (AST) >2× upper normal limit) or renal insufficiency (serum creatinine >200 micromol/L)
- Severe epilepsy with frequent tonic-clonic (grand mal) seizures
- Insulin treatment (type 1 diabetes)
- Active cancer disease requiring chemotherapy or radiation therapy
- Severe chronic disease (eg, cirrhosis and AIDS)
- Primary testosterone deficiency in the form of testicular dysgenesis, Klinefelter syndrome (47, XXY), 46, XX male, luteinizing hormone resistance, Y chromosome deletions, and other sex chromosomal abnormalities
- Severe mental retardation, dementia, or physical disabilities leading to inability to participate in the exercise intervention and the physical tests, or to give informed consent
- Contraindications for testosterone undecanoate treatment such as presence of liver tumors and breast carcinoma

Participants receiving resistance training also receive a total of 34 g of protein supplement immediately after each completed training session. Protein supplement has shown beneficial effect in participants aged 70 years [40], and participants are instructed to note this in a project diary. Additionally, participants are given a daily vitamin D supplement of 38 mg in combination with 800 mg calcium.

### Training Intervention

Four weeks after the first injection, participants in the training groups will receive progressive resistance training 3 times weekly (Monday, Wednesday, and Friday) for 16 weeks led by 1 or 2 physiotherapists in groups of up to 10 participants. Group size may vary depending on rate of inclusion and the random allocation of participants to training. Groups of up to 5 participants will be supervised by 1 physiotherapist, and an extra physiotherapist will be assigned to larger groups. A group of 5 physiotherapists with varying experience (2-17 years) led and instructed by a physiotherapist with extensive experience with progressive resistance training of patients with different diagnoses including older men with prostate cancer [41] will supervise the training sessions. The training takes place in the rehabilitation facilities at the hospital, where appropriate strength training equipment (Technogym, Gambettola, Italy) and stationary bikes (Monark model 828E and 927E, Vansbro, Sweden) are used.

### Motivation and Compliance

The supervising physiotherapists will continuously motivate the participants to perform the exercises with the intended intensity and try to facilitate an inspiring training environment and a sense of team spirit. Time and place for social interaction between participants before and after each training session are provided, and the participants are encouraged to engage.

A log book of attendance to training sessions is kept by the physiotherapists. The number of sets and repetitions performed for each exercise at every training session is noted by the participants and physiotherapists in collaboration, and close supervision of execution of the exercises is provided by the physiotherapists to ensure compliance with the planned program.

### Progressive Resistance Training Program

Participants start with a 10-min self-paced warm-up on a stationary bike. Patients with comorbidities that compromise stationary cycling, warm up on a rowing ergometer (Concept2 model D, Concept2, Morrisville, VT, USA). The warm up is followed by approximately 45 min of progressive resistance training on machines (Technogym, Element series) including the following specific machines: leg press, leg curl (hamstrings), leg extension (quadriceps), abdominal crunch, lower back, low row, and chest press. Adjustment of the machines is done by the supervising physiotherapist at the initial training session and followed up throughout the training period. The machines are adjusted to fit the individual participants aiming for full range of motion while accommodating any individual needs of the participants. The order of the exercises may vary from session to session. To induce adequate muscular fatigue and thus an adequate training stimulus, all sets of one exercise are completed with a 1- to 2-min rest in between sets before moving on to the next exercise. The intensity of the exercises is progressed by increasing the weight lifted and the number of sets performed while decreasing the number of repetitions in each set during the 16-week training period. The American College of Sports Medicine (ACSM) guidelines for progression are used for guidance [42] while carefully accommodating participant feedback and special needs and challenges of the individual participants. A slow progression beginning with 2×15 repetitions of each exercise with low load for the first 2 sessions is used to give the often relatively frail patients time to get familiarized with the machines, learn the correct execution of each exercise, and adapt to the training without experiencing excessive muscle soreness. The initial loading of each exercise is decided by the supervising physiotherapist in close cooperation with each individual participant. The targeted progression over the 48 planned training sessions is illustrated in Table 1. To ensure a proper progression in the training load in accordance with the program, the weight lifted in each exercise is continuously adjusted throughout the training period by the supervising physiotherapist. If a participant can perform significantly more (2-3 repetitions) than the planned number of repetitions in each set of a particular exercise, the loading is increased to reach the desired repetition maximum (RM)— that is, the number of repetitions that can be performed with proper technique before repetition failure. If one or more training sessions are missed, the loading will be adjusted by the supervising physiotherapist aiming for the number of repetitions in each set and the corresponding RM for the session when the participant returns in accordance with the progression plan in Table 1.

**Table 1:** Progression of resistance training over the 48 planned training sessions.

Sessions	Sets × repetitions	Intensity
1-2	2 × 15	20-25 RM^a^
3-6	2 × 12	12-15 RM
7-13	3 × 12	12-15 RM
14-48	3 × 10	10-12 RM

^a^RM: repetition maximum; the number of repetitions that can be performed with proper technique.

### Data Collection

A data collection procedure and a database are prepared for registration and data processing. Information regarding demographic and physical characteristics collected at baseline includes the following: age, single or cohabiting, help at home, mobility aids, social network, height, weight, waist-hip ratio, smoking habits, and alcohol consumption. Baseline testing as described below is performed before participants are randomized to one of the training groups, starting training on day 30. Patients are retested after 20 weeks, when training is discontinued. Participants receiving no training are tested in the same way and at the same time points as those who receive progressive resistance training.

### Primary Endpoint

The 30-second chair stand test is used to measure improvement of general strength, power, and endurance in lower extremities. The number of times the participant can rise from a chair in 30 seconds with arms crossed over the chest and returning to a sitting position between each repetition is counted by a test leader. Repetitions are only counted as successful if full extension of the hips and knees is achieved for each rise. The back does not need to touch the backrest of the chair when returning to a sitting position, but contact with the seat must be made. This test has a satisfactory correlation (r=.78) with leg press exercise abilities [43], and an acceptable test retest reliability (interclass correlation [ICC]=.86) has been found [44]. The 30-second chair stand test provides a reasonably reliable and valid measurement of lower body strength of older adults, and easily separates low-active participants from high-active participants [45]. For men aged 70 to 74 years, a 30-second chair stand test performance between 12 (25th percentile) and 17 (75th percentile) is considered normal; thus, we chose to only include older men in this trial with a performance of 12 or less [46]. It has recently been scientifically proven that the ability of elderly persons to perform a slightly different version (timing of 5 chair-stands) of this simple test correlates with the risk of serious fall injuries [47].

### Secondary Endpoints

#### Frequency and Severity of Adverse Events

This is recorded using a questionnaire for each subject and is included in the monitoring of adverse events.

#### The Mobility Scale of Avlund

The mobility scale of Avlund include questions about experienced fatigue and support needs in activities of daily living. The mobility scale of Avlund is correlated with isometric muscle strength, simple functions [48], increased risk of hospitalization [49], and mortality [50]. Inter-rater reliability values of kappa .72 to 1.00 have been shown [51].

#### Major Depression Inventory

This questionnaire will be used to estimate depression severity and mental well-being [52].

#### Montreal Cognitive Assessment

Montreal Cognitive Assessment is a cognitive screening test used to provide an estimate of the cognitive functions. This test is sensitive to mild cognitive problems and dementia [53].

#### Quality of Life EuroQol-5 Domain

This questionnaire is used to estimate experienced quality of life in participants [54].

#### Fatigue Severity Scale

This questionnaire is used to estimate fatigue among participants [55].

#### Pelvic Organ Prolapse/Urinary Incontinence Sexual Function Questionnaire-12

This questionnaire is used for evaluation of sexual function before and after treatment, including assessment of sexual ability and sexual desire. Improved sexual function, mood, muscle power, and body composition have been found in hypogonadal men treated with testosterone supplement [32].

#### Graded Cycling Test with Talk Test

This is a submaximal aerobic exercise test that will be used to measure potential changes in aerobic capacity [56,57].

#### Arm Flexion Test

This test measures general strength in upper extremities. The number of times in 30 seconds a participant can flex the elbow with a 3 kg weight in the hand is counted. The test has a satisfactory correlation (*r*=.81) with muscle strength of the biceps, chest, and upper back muscles and an acceptable test retest reliability (ICC=.81) [44].

#### Timed Up and Go Test

This test measures basic mobility in the elderly. The time taken to rise from a chair, walk 3 m away, and return to a sitting position in the chair is measured. A moderate to good correlation has been shown between this test and Berg's Balance Scale (*r*=.81), velocity (*r*=.61), and Barthel Index (*r*=.78) [38], and good test retest reliability has been observed (ICC=.98) [43]. The best of three attempts is recorded [58].

#### Dual-Energy X-ray Absorptiometry Scanning and Bioimpedance Measurement of Body Composition

Dual-energy x-ray absorptiometry (DXA) scanning is used to measure lean body mass corresponding to measurements of fat and fat-free mass as well as total bone mass. DXA is performed as whole-body scan at baseline and at the end of the study.

Bone mineral density in the columnar, bilateral distal forearm, total hip bilaterally, and overall skeleton is measured. If osteoporosis is detected, treatment will be initiated according to the department's usual guidelines after the participant has finished trial participation.

These measurements are performed as fasting morning measurements. If fasting is impossible or extremely difficult, a standardized breakfast will be used. It is expected that the fat-free mass will increase and that the fat percentage will be reduced from baseline to the end of training. In addition, a further improvement of the aforementioned variable in the active treatment group is expected, thus documenting an additive effect of the combination of exercise, protein supplements, and testosterone substitution. The two methods for estimating body compositions over time will be compared. Importantly, each test result at the end of the trial is compared with the similar test result obtained at baseline, so that changes over time can be measured for each participant. This approach has been used in several publications [59,60].

### Safety Parameters Measured Before Every Injection of Placebo or Testosterone Undecanoate

Blood pressure is checked, and blood tests are performed for the following: serum testosterone, hemoglobin, hematocrit, lipid profile, potassium, sodium, creatinine, C-reactive protein, aspartate aminotransferase, bilirubin, alkaline phosphatases, thyroid-stimulating hormone, ionized calcium, parathyroid hormone, Ca^2+^, and 25-OH vitamin D. Before and during treatment with testosterone undecanoate, prostate palpation is performed to examine for prostate cancer. Subjects with enlarged prostate or irregular prostate surface are examined by a urologist and may be considered for prophylactic treatment against benign prostatic hyperplasia with Finasteride, which suppresses prostate hyperplasia with testosterone supplementation without side effects [34]. In addition, adverse reactions and adverse events are recorded very carefully, for example, in the form of falls and fall severity, ischemic episodes, and the like.

Blood samples are not stored for more than 1 week and are not included in a research biobank. The biological material is analyzed immediately and destroyed afterward. No part of the blood samples will be used in personally identifiable ways.

### Sample Size Estimation

The primary endpoint is to improve 30-second chair stand test performance [35]. According to normative scores, a performance of 15 is considered normal (50th percentile) for older men aged 70 to 74 years, whereas a performance of 11 or less is considered abnormal [46]. Our main hypothesis is that controls will achieve a performance in the 30-second chair stand test performance of 11 (64% of the 75th percentile), whereas the combination group will achieve a performance of 15 (88% of the 75th percentile). If an alpha value of .05 and a beta value of .2 are used; the needed sample size is 38 participants in each group. Due to the high age and time-consuming intervention, up to 25% dropouts are expected, and we therefore need to include 48 participants in each group.

### Statistical Analysis

The results will be analyzed by intent-to-treat and per protocol for participants who have followed 60% of testosterone treatment and exercise. Regarding statistical data processing, data will be ranked and group comparisons will be performed by nonparametric tests (Kruskal-Wallis and Mann-Whitney). Comparisons of ranks at baseline and at the end of the study period are performed using Wilcoxon nonparametric test and optionally categorically variable with chi-square test. Nonlinear correlations will be evaluated using the Spearman rank correlation coefficient. Untrusted or unused data are not included in the statistical evaluation and will be treated as missing data, that is, no calculations are made using such data. Lack of data is acceptable as long as an assessment of the primary endpoint is still possible. Thus, data from subjects that make it possible to assess the primary endpoint will be used as a minimum; however, we hope that data from all randomized patients can be used to evaluate secondary effect variables. *P* values of less than .05 will be considered significant.

### Ethics

The project complies with the Helsinki Declaration. The trial is approved by the Danish Data Protection Agency, and the trial protocol has been approved by the relevant scientific ethics committee, protocol number: H-16020521, and can be initiated. ClinicalTrials.gov Identifier: NCT02873559.

In the trial, only patients with testosterone deficiency are treated; thus, testosterone undecanoate is used according to indication and in compliance with recommendations by the Danish Medicines Agency. The following section describes possible side effects and risks. We do not expect significant adverse reactions from participants; treatments may primarily result in improved motor skills, reduced fall risk, improved cognitive skills, reduced risk of cardiovascular disease, improved quality of life, better sexual function, and lower risk of developing depression. The project is focused on normalizing and increasing both health and quality of life. We are convinced that the benefits of the treatments in this trial by far outweigh the risks of adverse events.

### Adverse Events and Risks

Strength training can cause delayed onset muscle soreness following the first training session. The mild soreness is temporary and diminishes as the muscles quickly adapt to the training. There are no serious side effects related to strength training. There are no side effects to protein supplements [19] and vitamin D [23]. Common (1-10%) adverse reactions to testosterone undecanoate include discomfort at the injection site, weight gain, elevated hematocrit, elevated hemoglobin, polycythemia, hot flushes, acne, elevated prostate-specific antigen, and prostate hypertrophy. Uncommon (0.1%-1%) adverse reactions include pain, dyspnea, hypertension, cardiovascular events, gynecomastia, hypercholesterolemia, hypertriglyceridemia, arthralgia, depression, mood disorders, dizziness, tremor, alopecia, erythema, hypersensitivity, lower respiratory tract infections, and urinary retention. Rare (0.01%-0.1%) adverse reactions include priapism. Very rare (<0.01%) adverse reactions include liver changes.

Cases of increased libido will be registered and will be addressed by the primary investigator who has many years of experience working with geriatric populations.

### DXA Scan

The participant must be able to lie supine for 15 min and will be in constant contact with personnel. No contrast materials are used. The scanner emits X-rays, which are harmful when exposed to larger doses. In this study, participants receive a small amount of radiation, corresponding to approximately 6 days of background radiation. In one scan, the risk of developing a life-threatening cancer increases from 25% (because of the background radiation) to 25.00025% — and to 25.00050% for two scans. Dropouts in both groups are noted with cause. Adverse events are recorded.

## Results

Funding was provided in October 2016. Enrollment was initiated in November 2016, and the first patient was included in January 2017. In January 2018, about 50 participants were included. Results are expected to be available in 2020.

## Discussion

### Overview

To the best of the authors’ knowledge, this is the first time an investigation will evaluate the effects of testosterone supplemental therapy and progressive resistance training on cognition, physical well-being, and quality of life. We have received all necessary funding. Our broad spectrum of secondary endpoints ensures a thorough examination of therapeutic benefits, beyond previous investigations. Furthermore, unlike previous studies, participants in this study will be older and have verified hypogonadism.

High-quality, randomized trials are lacking in older men, and therefore in this trial, a diagnosis of hypogonadism is based on the presence of both clinical symptoms and low serum testosterone levels similar to diagnosing hypogonadism in younger or middle-aged men [61]. To complicate hypogonadism diagnosis further, other symptoms caused by aging may overlap with symptoms of hypogonadism. We are aware that using serum testosterone levels ≤13 nmol/L may cause an overdiagnosis of hypogonadism, because many older men with low testosterone are asymptomatic. To avoid treating potentially asymptomatic older men, in this trial, we only include older men with low testosterone experiencing reduced physical abilities. It may be noted that this trial does not primarily focus on improving serum testosterone, but focuses on improving overall physical and mental abilities of older men, where serum testosterone is one of the several potential key components.

Testosterone supplements have previously been suspected of contributing to cardiovascular disorders, but a recent investigation based on 7245 men did not find any correlation between testosterone supplements and cardiovascular disorders [62]. Furthermore, investigators performing a major meta-analysis based on 122,889 participants supported these findings [63]. It should be noted that the mean age of the study with 7245 men was 54 years; thus, most men were not among the elderly, and few octogenarians if any may have been included. The study of 122,889 men currently has been published as an abstract without any description of the mean age of participants. Thus, it is not known if results obtained from such huge materials are relevant for older men.

Previously, it has also been found that testosterone deficiency is a risk factor for atherosclerosis, myocardial infarction, and chronic heart failure [26-28]; thus, we believe that the overall health of participants receiving testosterone supplemental therapy combined with physical resistance therapy will be significantly improved through this trial.

### Limitations

Finding eligible patients may be difficult, and our inclusion and exclusion criteria limit our investigation to frail older men still able to walk unassisted. Our results will not include patients with a more profound need of rehabilitation and treatment.

### Conclusions

This trial will evaluate the effects of testosterone supplemental therapy alone or combined with progressive resistance training. By using a broad spectrum of tests, we aim to provide a clear answer to whether or not such intervention may benefit frail older men.

## Abbreviations

DXA: dual-energy x-ray absorptiometry
ICC: interclass correlation
RM: repetition maximum
